# Patient selection for corneal topographic evaluation of keratoconus: A screening approach using artificial intelligence

**DOI:** 10.3389/fmed.2022.934865

**Published:** 2022-08-04

**Authors:** Hyunmin Ahn, Na Eun Kim, Jae Lim Chung, Young Jun Kim, Ikhyun Jun, Tae-im Kim, Kyoung Yul Seo

**Affiliations:** ^1^Department of Ophthalmology, The Institute of Vision Research, Yonsei University College of Medicine, Seoul, South Korea; ^2^Eyejun Ophthalmic Clinic, Seoul, South Korea

**Keywords:** artificial intelligence, corneal topography, keratoconus, machine learning, Pentacam, screening test

## Abstract

**Background:**

Corneal topography is a clinically validated examination method for keratoconus. However, there is no clear guideline regarding patient selection for corneal topography. We developed and validated a novel artificial intelligence (AI) model to identify patients who would benefit from corneal topography based on basic ophthalmologic examinations, including a survey of visual impairment, best-corrected visual acuity (BCVA) measurement, intraocular pressure (IOP) measurement, and autokeratometry.

**Methods:**

A total of five AI models (three individual models with fully connected neural network including the XGBoost, and the TabNet models, and two ensemble models with hard and soft voting methods) were trained and validated. We used three datasets collected from the records of 2,613 patients' basic ophthalmologic examinations from two institutions to train and validate the AI models. We trained the AI models using a dataset from a third medical institution to determine whether corneal topography was needed to detect keratoconus. Finally, prospective intra-validation dataset (internal test dataset) and extra-validation dataset from a different medical institution (external test dataset) were used to assess the performance of the AI models.

**Results:**

The ensemble model with soft voting method outperformed all other AI models in sensitivity when predicting which patients needed corneal topography (90.5% in internal test dataset and 96.4% in external test dataset). In the error analysis, most of the predicting error occurred within the range of the subclinical keratoconus and the suspicious D-score in the Belin-Ambrósio enhanced ectasia display. In the feature importance analysis, out of 18 features, IOP was the highest ranked feature when comparing the average value of the relative attributions of three individual AI models, followed by the difference in the value of mean corneal power.

**Conclusion:**

An AI model using the results of basic ophthalmologic examination has the potential to recommend corneal topography for keratoconus. In this AI algorithm, IOP and the difference between the two eyes, which may be undervalued clinical information, were important factors in the success of the AI model, and may be worth further reviewing in research and clinical practice for keratoconus screening.

## Introduction

Keratoconus is a chronic, progressive, non-inflammatory corneal disorder where the central or paracentral cornea undergoes thinning in individuals aged 18–40 years (1). While the disease course varies depending on its progression, keratoconus may eventually require a treatment as extensive as corneal transplantation due to corneal scarring and perforation. According to a previous meta-analysis, the prevalence of keratoconus in the study population was 1.38 per 1,000 persons (2). On the other hand, in a previous nationwide population-based study, the incidence of keratoconus was 5.56 per 100,000 (3). However, these hospital-based epidemiological reports should be interpreted with caution since the true prevalence or incidence of keratoconus within the general population may be underestimated (4).

Slit-lamp examination and corneal topography/tomography are important tests performed to help detect and manage keratoconus (1). In particular, corneal topography is not only diagnostic for keratoconus, but also provides information regarding its severity and progression. To date, most of the studies investigating keratoconus have been based on topographic results (5–8). Unfortunately, corneal topography is not routinely performed for patients visiting the clinic, including first-time patients, unlike the tests for visual acuity and intraocular pressure (4, 9). Moreover, the Medicare Advantage Policy Guidelines outline a list of indications for corneal topography that limits routine testing (10). For these reasons, keratoconus is usually detected through other ways. For example, advanced keratoconus may be detected by an ophthalmologist after failing for vision correction with glasses due to severe corneal astigmatism or corneal scar. Alternatively, detailed preoperative examinations for corneal refractive surgery or cataract surgery may reveal keratoconus or its latent form, even in asymptomatic patients. As a result, when keratoconus is detected, it is often by chance and may already be at an advanced stage that ultimately requires a corneal transplantation. If it is possible to identify patients who need corneal topography with only ophthalmologic examinations, performed basically for patients who visit eye clinics, keratoconus may be diagnosed in a latent or early stage before visual impairment.

Artificial intelligence (AI) is widely studied in the medical field, and ophthalmology is no exception. For keratoconus, the ability for AI to detect keratoconus was excellent with over 90% accuracy (11). Since the invention and development of corneal topography and its analysis program, keratoconus has been a disease that can be early diagnosed through corneal topography. However, there is a lack of research on how to select patients who would best benefit from corneal topography for keratoconus screening. Currently, one of the biggest problems associated with keratoconus detection is that patients who need corneal topography are not preemptively identified. To solve this problem, our study aimed to develop a tool using a novel AI that recommends corneal topography for keratoconus screening based on the results of basic ophthalmologic examinations. Furthermore, the clinical factors that affected the estimation of AI were analyzed by the feature importance of explainable AI.

## Materials and methods

### Study design and population

**Figure 1:**
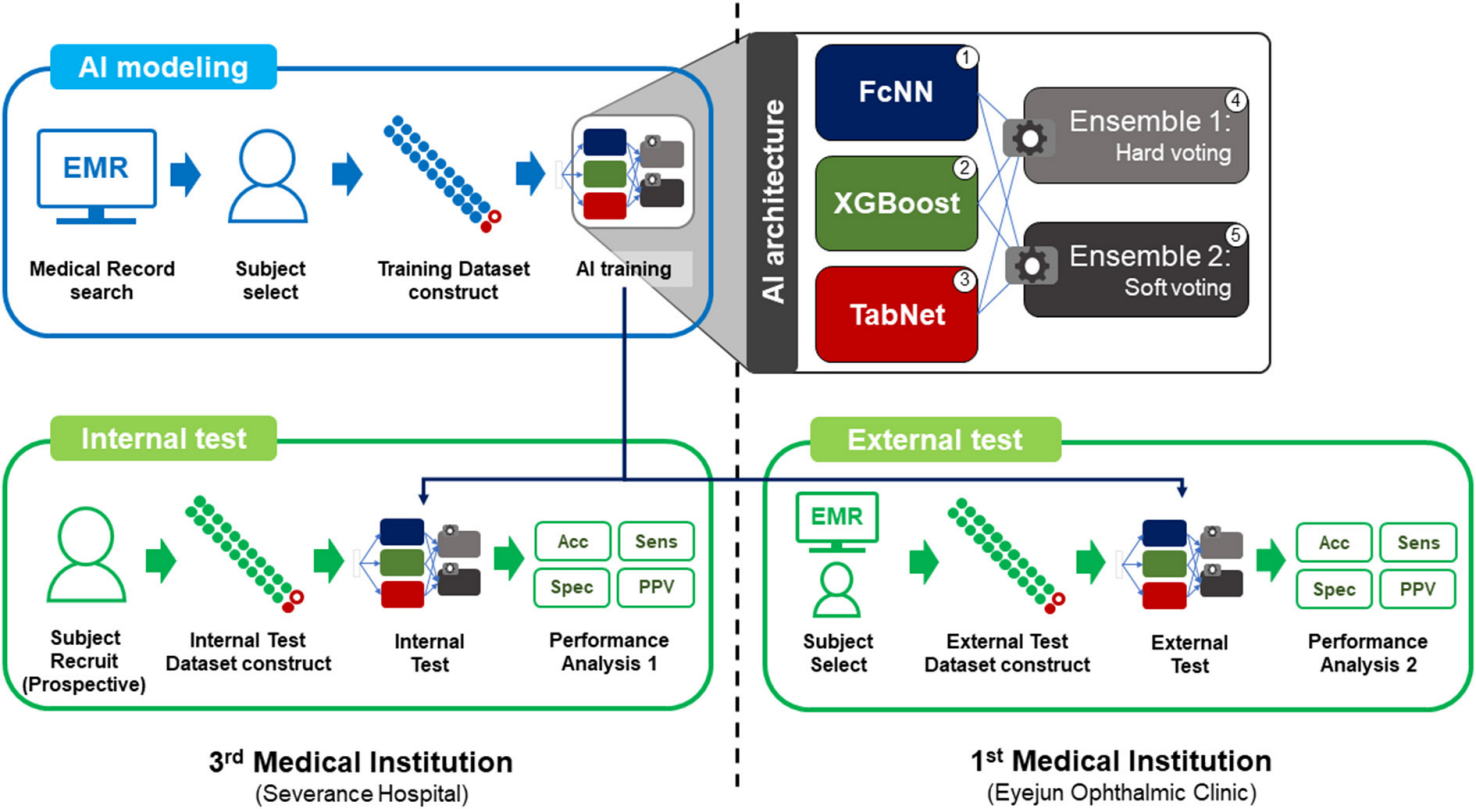
Overview of the study.

This study was conducted with three datasets from two medical institutions, including a tertiary medical institution (Severance Hospital, Yonsei University College of Medicine) and a primary medical institution (Eyejun Ophthalmic Clinic) (Figure 1). First, we retrospectively extracted data for AI model training from the medical records of individuals who first visited Severance Hospital from July 2015 to August 2021 (training dataset). We then prospectively recruited study participants who first visited Severance Hospital between September 2021 and November 2021 (internal test dataset). For extra-institutional validation, we used the data from individuals who first visited Eyejun Ophthalmic Clinic between January 2021 and September 2021 (external test dataset). The study was conducted in accordance with the tenets of the Declaration of Helsinki, and ethical approval for each follow-up was obtained from the Institutional Review Board of Yonsei University College of Medicine. All participants for prospective validation provided written informed consent before participating. For retrospective data, the subject consent exemption was granted after IRB approval (Protocol number 1-2021-0054 and 4-2022-0326).

All study subjects completed a medical interview and basic ophthalmologic examinations including visual acuity test, tonometry, slit-lamp examination, and corneal topography during their first visit. Patients with a history of eye trauma or eye surgery, such as corneal refractive surgery or cataract surgery, were excluded. Furthermore, patients who were followed up prior to 6 months were excluded.

### Clinical assessments

During the medical interview, patients described their symptoms as progressive, consistent, and/or uncorrected visual impairment. Basic ophthalmologic examinations included those that can be routinely performed at each visit, including manifest refraction to measure best corrected visual acuity (BCVA), non-contact tonometry (NCT) to measure intraocular pressure (IOP), and autokeratometry. When IOP was under 7 mmHg or over 21 mmHg, measurements were repeated twice more. When the initial measurement of refraction or keratometric power by autokeratometry failed, a repeat measurement was performed. Slit-lamp examination was performed by experienced corneal specialists to evaluate the corneal sequelae of keratoconus, including corneal thinning, scarring, and hydrops. Corneal topography was measured by Pentacam (Oculus, Wetzlar, Germany) imaging. The quality of each Pentacam image was reviewed by the operator at the time of image acquisition and repeated if required. Only data from images that displayed a quality specification (an internal, automatic assessment of imaging quality) of “OK” were included in this analysis (12).

### Clinical and subclinical keratoconus

Topographic evidence of keratoconus was confirmed by Pentacam, and both of following criteria were satisfied: (1) abnormal localized steepening or an asymmetric bow-tie pattern in tangential map and (2) D-score (BAD-D) ≥2.6 (pathologic) or ≥1.6 (suspicious) in Belin-Ambrósio enhanced ectasia display (13, 14). A BAD-D has a very low false-positive rate (15). Topographic keratoconus progression was confirmed when the mean corneal power in the steepest meridian (K2) or maximum keratometry in the steepest corneal power (Kmax) increased two or more consecutive times with each 3-month follow-up interval (16, 17). Clinical keratoconus was confirmed if one of following criteria with topographic evidence was satisfied: (1) Abnormal finding of keratoconus in slit-lamp examination, (2) symptomatically progressive visual impairment with BCVA under 0.8 decimal scale, (3) topographic keratoconus progression, (4) clinical keratoconus in the contralateral eye. Subclinical keratoconus was confirmed as follows: (1) only topographic evidence exist in both eyes, (2) no topographic evidence of keratoconus but clinical keratoconus in the contralateral eye (5).

### Artificial intelligence modeling

Python version 3.8 was used for AI development and statistical analysis. The following information from both eyes for an individual subject were considered as input variables: (1) presence of visual impairment (2) BCVA, (3) IOP, (4) autokeratometry values including the refractive power [sphere (Sph), cylinder (Cyl), cylinderic axis (Ax)], and the flatter (K1) and steeper (K2) corneal power and K2 axis (K2ax), (5) mean corneal power (Km, (K1+K2)/2) and corneal astigmatism (Kast, K2-K1), and (6) differences in BCVA, IOP, spherical equivalent (SE, sphere+cylinder/2), Km, and Kast between the right and left eyes. When one or both eyes of an individual patient was considered to have clinical or subclinical keratoconus, we confirmed that corneal topography is recommended, and the recommendation of corneal topography was set as the output variable.

The AI model was developed with five types of AI architecture, with three individual architectures including: (1) a 5-layer fully connected neural network (FcNN) with L2 regularization and dropout 0.5, (2) the XGBoost (18), and (3) the TabNet (19). Two ensemble models of these individual architectures were also used, including: (4) “Ensemble model 1” using the ensemble method of “hard voting,” which predicts the class with the largest sum of votes from models, and (5) “Ensemble model 2” using the ensemble method of “soft voting,” which predicts the class with the largest summed probability from models (Figure 1).

Keratoconus has a low prevalence, and imbalance between classes can bias the model used for AI training and validation (20). For our training dataset, we first extracted information pertaining to the subjects who were recommended corneal topography. Next, we randomly extracted information of twice as many normal subjects as those who were recommended corneal topography, by age-matched down-sampling using Python.

For duplicate IOP data, the lowest value was considered because inaccurately high readings of NCT measurement are frequent in poorly compliant patients if they squeeze their eyelids (21). The unmeasured values of autokeratometry were converted to the highest possible values available on the autokeratometry instruments.

### Statistical analysis

To evaluate the performances of AI architectures, four parameters including accuracy, sensitivity, specificity, and positive predictive value (PPV) were calculated. Error rate was defined as (1-accuracy). In the internal and external tests, the error rates of individual AI models were evaluated according to the BAD-D and the classes of normal, subclinical keratoconus, and clinical keratoconus on a single eye. For explainable AI modeling, feature (input variable) importance of each single model was analyzed. Feature importance was evaluated with feature attribution of input variable. The Shapley (SHAP) value in the FcNN model (22), built-in F-score in the XGBoost model (18), and built-in feature attribution in the TabNet model were used (19). The average value of right and left eyes was used for a single feature. Relative feature attribution was calculated as a value relative to the maximum value of feature attribution in each AI model. Features were ranked according to relative feature attributions. The average values of ranks and feature attributions in the features were calculated and ranked for feature importance.

## Results

Overall demographics and characteristics of the study population are described in Table 1. For the AI training dataset, 91,367 patients were screened and 1,518 patients were selected. Among them, 34.19% were subjects for whom corneal topography was recommended. A total of 457 patients were enrolled in the prospective internal test, and 16.19% of them were recommended corneal topography. A total of 638 patients were selected in the dataset for external validation, and 17.40% of them were recommended corneal topography.

**Table 1:** Demographics and characteristics of study population.

	Training dataset^*^	**Internal test dataset**	**External test dataset**
Sreening subjects	91,367	1,556	51,447
Recruit subjects	1,518	457	638
**Diagnosis**			
Normal (%)^†^	999 (65.81)	383 (83.81)	527 (82.60)
Subclinical keratoconus (%)	69 (4.55)	38 (8.32)	43 (6.74)
Clinical keratoconus^‡^ (%)	450 (29.64)	36 (7.88)	68 (13.66)
Recommend corneal	519 (34.19)	74 (16.19)	111 (17.40)
topography (%)			
**Demographics**			
Age, years (mean ± SD)	34.43 ± 12.04	26.54 ± 8.58	28.26 ± 12.52
Sex			
Female (%)	789 (51.98)	236 (51.64)	330 (51.72)
Male (%)	729 (48.02)	221 (48.36)	308 (48.28)
**Subjective visual impairment**			
Right (%)	482 (31.75)	27 (5.91)	68 (10.61)
Left (%)	503 (33.14)	22 (4.81)	67 (10.50)
**Basic examinations**			
BCVA, logMAR			
Right (mean ± SD)	0.265 ± 0.431	0.056 ± 0.199	0.092 ± 0.256
Left (mean ± SD)	0.243 ± 0.379	0.053 ± 0.183	0.085 ± 0.258
IOP, mmHg			
Right (mean ± SD)	12.12 ± 4.52	15.78 ± 3.24	14.90 ± 3.89
Center (mean ± SD)	11.98 ± 3.67	15.89 ± 3.23	14.92 ± 3.76
**Autokeratometry**			
Refrective errors			
Sphere, diopter			
Right	−3.78 ± 3.76	−2.51 ± 3.07	−2.60 ± 3.04
Left	−3.84 ± 3.85	−2.36 ± 3.00	−2.35 ± 3.44
Cylinder, diopter			
Right	−2.94 ± 2.30	−1.43 ± 1.57	−1.81 ± 1.91
Center	−3.14 ± 2.78	−1.49 ± 1.60	−1.91 ± 1.73
Cylinderic axis, degree			
Right	87.99 ± 68.30	97.10 ± 71.63	91.02 ± 72.14
Left	114.56 ± 63.25	102.93 ± 67.44	109.80 ± 66.22
Corneal power			
K1 (flatter), diopter			
Right	43.70 ± 4.40	42.59 ± 2.11	43.30 ± 2.59
Left	43.27 ± 6.53	42.54 ± 1.94	43.30 ± 2.67
K2 (steeper), diopter			
Righht	46.76 ± 5.72	44.25 ± 2.98	44.99 ± 4.60
Left	46.74 ± 5.79	44.18 ± 2.71	45.32 ± 3.67
K2 axis, degree			
Right	97.226 ± 32.19	86.05 ± 26.52	89.25 ± 21.41
Left	81.02 ± 33.00	91.31 ± 25.81	88.57 ± 25.01
**Corneal topography**			
Abnormal in tangenital map^§^			
Right (%)	513 (33.79)	73 (15.97)	116 (18.18)
Left (%)	496 (32.67)	73 (15.97)	116 (18.18)
D-score in BAD			
Right (mean ± SD)	2.14 ± 0.67	1.65 ± 0.33	1.80 ± 0.41
Pathologic (%)	431 (28.39)	35 (7.66)	81 (12.70)
Left (mean±SD)	2.13 ± 0.67	1.65 ± 0.33	1.80 ± 0.41
Pathologic (%)	421 (27.73)	30 (6.56)	83 (13.01)

*BCVA, best-corrected visual acuity; BAD, Belin/Ambrósio enhanced ectasia display; SD, standard deviation*.

* *Down sampling method was adjusted for normal subjects*.

† *Normal in both eyes*.

‡ *Clinical keratoconus in at least one eyes*.

§ *Abnormal localized steepening or an asymmetric bow-tie pattern*.

For the performance of the AI models, in the training dataset, the XGBoost and the Ensemble 1 models showed the highest accuracy of 94.7% (Table 2). Sensitivity was highest in the TabNet and the Ensemble 2 models (both 98.5%). In the internal test dataset, the Ensemble 1 model showed the highest accuracy and specificity of 95.6 and 97.9%, respectively. Sensitivity was highest in Ensemble 2 (90.5%). In the external test dataset, the XGBoost model showed the higest accuracy, specificity, and PPV of 90.1, 90.1, and 65.8%, respectively. Sensitivity was highest in the Ensemble 2 model (96.5%).

**Table 2:** Performances of artificial intelligence models.

	FcNN^*^	**XGBoost**	**TabNet**	**Ensemble 1^**†**^**	**Ensamble 2^**‡**^**
**A. Training dataset**					
Accuracy	0.943	**0.947**	0.875	**0.947**	0.870
Sensitivity	0.934	0.944	**0.985**	0.977	**0.985**
Specificity	**0.948**	**0.948**	0.819	0.932	0.811
PPV	0.903	0.738	**0.904**	0.882	0.730
**B. Internal test dataset**					
Accuracy	0.930	0.947	0.906	**0.956**	0.934
Sensitivity	0.676	0.784	0.892	0.838	**0.905**
Specificity	**0.979**	**0.979**	0.909	**0.979**	0.940
PPV	0.862	**0.906**	0.653	0.886	0.744
**C. External test dataset**					
Accuracy	0.875	**0.901**	0.856	0.893	0.854
Sensitivity	0.757	0.901	0.919	0.856	**0.964**
Specificity	0.899	**0.901**	0.843	**0.901**	0.831
PPV	0.613	**0.658**	0.551	0.646	0.546

*FcNN, fully connected neural network; PPV, positive predictive value*.

* *A 5-layered fully connected neural network with L2 regularization and dropout 0.5*.

† *Hard voting ensamble method with FcNN, XGBoost, and TabNet*.

‡ *Soft voting ensamble method with FcNN, XGBoost, and TabNet*.

*Bold values indicate the best result of the individual performance (row)*.

**Figure 2:**
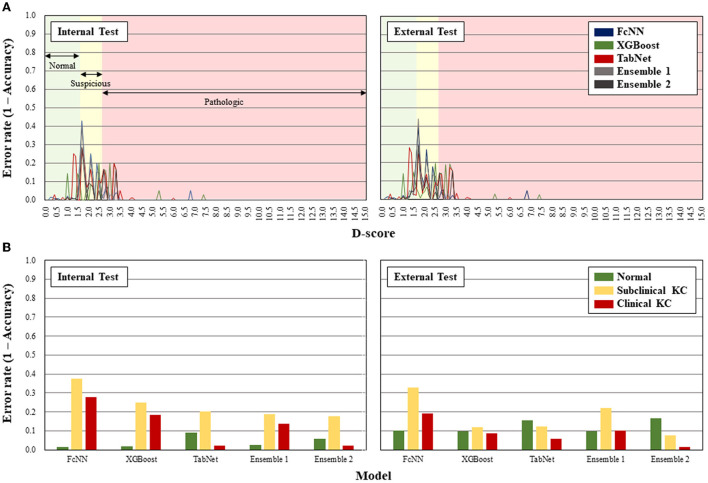
Error rates of the artificial intelligence models according to D-score of Belin-Ambrósio enhanced ectasia display **(A)** and classification of subclinical and clinical keratoconus **(B)**.

For error analysis, in the internal test, the error rates within the suspicous BAD-D range were 37.5, 25.0, 20.4, 18.8, and 17.6%, respectively, and those within the pathologic BAD-D range were 27.9, 18.6, 2.3, 13.9, and 2.3% in the FcNN, XGBoost, TabNet, Ensemble 1, and Ensemble 2 models, respectively (Figure 2). In the external test, the error rates within the suspicous BAD-D range were 33.0, 12.0, 12.3, 22.1, and 7.6%, and those within the pathologic BAD-D range were 19.1, 8.8, 5.9, 10.3, and 1.5%, respectively. Over 95% of errors occurred under 3.5 range of BAD-D. Subclinical keratoconus showed the highest error rate in most of the AI models except the TabNet and Ensemble 2 models in the external test dataset. The Ensemble 2 model showed the lowest error rate in detecting both subclinical and clinical keratoconus.

**Figure 3:**
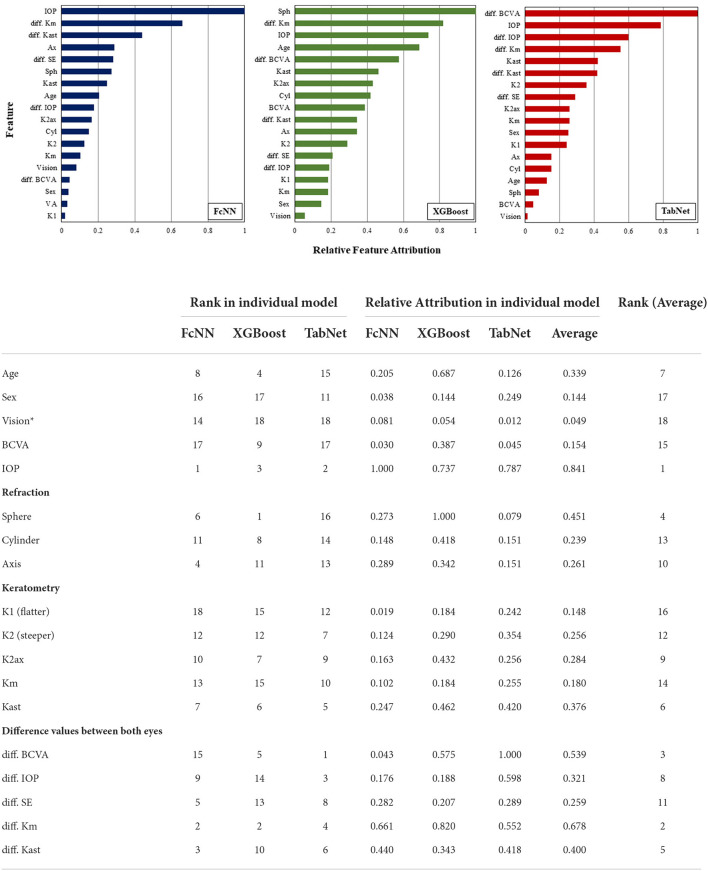
The explainable interpretation of feature importances in the individual AI models. *Symptoms as progressive, consistent, and/or uncorrected visual impairment. BCVA, best-corrected visual acuity; IOP, intra-ocular pressure; Km, mean value of corneal power; Kast, corneal astigmatism; SE, spherical equivalent.

For feature importance analysis, IOP was ranked highest followed by the difference in Km in the average value of the feature attributions (Figure 3). The five difference values between both eyes were positioned up to rank 8 out of 18 features in the TabNet model, which had higher sensitivity than the FcNN and XGBoost models.

## Discussion

In this study, we developed an AI model to identify patients for keratoconus screening by corneal topography, based on results of basic ophthalmologic examinations. The Ensemble 2 model with soft-voting ensemble method showed superior sensitivity compared with the other AI models in screening for keratoconus. This result was consistent with those of the internal prospective validation and external validation performed at the primary medical institution with a different patient population.

The most important consideration when designing this study was whether it is possible to identify patients who need corneal topography for keratoconus by using basic ophthalmologic examinations that are commonly performed in the clinical setting. Despite aiming to detect both clinical and subclinical keratoconus, the AI models performed fairly well. Although the error analysis suggested that the range of suspicion of BAD-D and subclinical keratoconus were the main error interval of this AI model, considering that it is difficult to predict keratoconus without corneal topography in this interval (13), and, in the local regression analysis of our training dataset (locally weighted smoothing [LOESS], α = 0.5, λ = 1) (23), BCVA started to deteriorate from BAD-D 3.6 and was worse than 0.1 logMAR from BAD-D 5.6 (Supplementary Figure 1), the AI model is expected to effectively screen for keratoconus. Moreover, in the case of clinical keratoconus, the screening accuracy was excellent, especially by the Ensemble 2 model. Corneal topography is essential to confirm the shape of the cornea to diagnose subclinical and mild cases of keratoconus; the AI model may facilitate early detection and treatment by selecting patients suspected of keratoconus before visual impairment based on the results of basic ophthalmologic examinations, rather than late, incidental detection often associated with advanced state of the disease.

Unlike traditional statistical analyses methods such as the linear regression analysis, AI analyzes the variability of individual elements through the reduction/increase and distortion of dimensionality expressed as a single variable (24). This AI mechanism accompanies problems such as vanishing gradient or curse of dimensionality (25, 26), but may also provide a solution to multi-factorial questions that are difficult for humans to recognize. Interpretation of high-performance AI can be difficult because it is unintuitive. Nevertheless, by using the analysis of feature importance/attribution, we can determine which value the AI considers to have the greatest weight in solving a problem (27–29). In this study, IOP showed the highest feature attribution. In keratoconus, corneal refractive power and astigmatism are expected to increase in autokeratometry, visual acuity may also worsen with disease progression (30), and corneal hysteresis and thickness, which affect IOP measurements, are also changed (31, 32). The present study shows that IOP was a more dominant feature in early keratoconus screening compared to BCVA and the autokeratometry results of which numerical changes were not clearly determined, implying that it may be worthwhile reconsidering the importance of factors other than morphological changes in examining clinical findings of keratoconus. Moreover, the difference in examination values between the eyes were also important features of this study. The patterns of progression of keratoconus in the two eyes are usually different (33), and the results of this study suggest that the difference between the right and left eyes is important in screening for the disease. Considering factors such as IOP and the relationship between the eyes will be important in future studies to predict the progression or examine prognostic factors of keratoconus.

A strength of this study is that only basic ophthalmologic examinations were used to develop an AI model to recommend corneal topography for detection of both clincial and subclinical keratoconus. Additionally, extra-validation confirmed that this method could be applied in primary medical settings. However, our study had some limitations. First, the AI was validated only in Korean populations. Therefore, further studies involving other ethnic groups are needed. Second, although the AI models, especially the Ensemble 2 model, showed high sensitivity of more than 90%, which is enough to be applicable in the actual clinical field, it exhibited relatively low PPV; therefore, a cost-effectiveness analysis may be required for its application for general health check-ups, where keratoconus is not as prevalent as in the hospital setting.

This study is the first to develop an AI system to recommend corneal topography for keratoconus detection based on basic ophthalmic examinations. An external evaluation with a different patient group demonstrated a sensitivity of over 90%. This study demonstrates a method by which early diagnosis and better prognosis may be achieved by recommending corneal topography in patients who may be affected by keratoconus. Furthermore, our findings highlight the utility of AI to reconsider the role of examinations that may have been underestimated for keratoconus detection in clinical practice.

## Data availability statement

The raw data supporting the conclusions of this article will be made available by the authors, without undue reservation.

## Ethics statement

The studies involving human participants were reviewed and approved by the Institutional Review Board of Yonsei University College of Medicine. The patients/participants provided their written informed consent to participate in this study.

## Author contributions

HA and KS designed the study and wrote the original draft of the manuscript. HA and NK collected patient data. JC, YK, IJ, T-iK, and KS provided resources. HA performed the artificial intelligence modeling and analysis. All authors provided critical review and approved the version for publication.

## Funding

This work was supported by Korea Mouse Phenotyping Project (NRF-2013M3A9D5072551) from the Ministry of Science and ICT through the National Research Foundation.

## Conflict of interest

The authors declare that the research was conducted in the absence of any commercial or financial relationships that could be construed as a potential conflict of interest.

## Publisher's note

All claims expressed in this article are solely those of the authors and do not necessarily represent those of their affiliated organizations, or those of the publisher, the editors and the reviewers. Any product that may be evaluated in this article, or claim that may be made by its manufacturer, is not guaranteed or endorsed by the publisher.

## Supplementary material

Supplementary Figure 1 The scatter plot demonstrating the relationship between the D-score of Belin-Ambrósio enhanced ectasia display and best-corrected visual acuity. The fitting curve of local regression (red solid line, α = 0.5, λ = 1) shows that BCVA started to deteriorate from BAD-D 3.6, and was worse than 0.1 logMAR from BAD-D 5.6.
