# Supplementary figures and images for: Patient selection for corneal topographic evaluation of keratoconus: A screening approach using artificial intelligence
Source: Front Med (Lausanne). 2022 Aug 4;9:934865. doi: 10.3389/fmed.2022.934865 (PMC9386450; doi:10.3389/fmed.2022.934865)

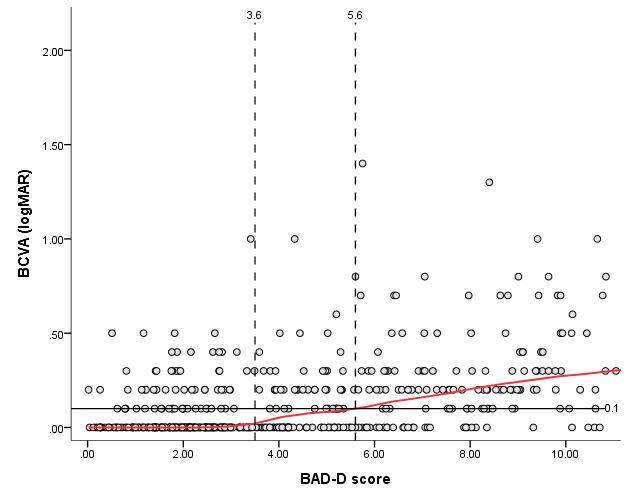

Supplement: Supplementary Figure 1 — The scatter plot demonstrating the relationship between the D-score of Belin-Ambrósio enhanced ectasia display and best-corrected visual acuity. The fitting curve of local regression (red solid line, α = 0.5, λ = 1) shows that BCVA started to deteriorate from BAD-D 3.6, and was worse than 0.1 logMAR from BAD-D 5.6. [file Image_1.PNG]
